# Quantitative urinary proteome analysis reveals potential biomarkers for disease activity of Behcet’s disease uveitis

**DOI:** 10.1186/s12886-024-03557-9

**Published:** 2024-07-09

**Authors:** Weiwei Qin, Anyi Liang, Xiaoxu Han, Meifen Zhang, Youhe Gao, Chan Zhao

**Affiliations:** 1 Department of Anesthesiology, Qingdao Hospital, University of Health and Rehabilitation Sciences (Qingdao Municipal Hospital), Qingdao, 266071 China; 2 Beijing Key Laboratory of Gene Engineering Drug and Biotechnology, College of Life Sciences, Beijing Normal University, Beijing100875, China; 3 Key Laboratory of Ocular Fundus Diseases, Department of Ophthalmology, Peking Union Medical College Hospital, Chinese Academy of Medical Sciences & Peking Union Medical College, Beijing, 100005 China; 4 Department of Ophthalmology, Guangdong Provincial People’s Hospital, Guangdong Academy of Medical Sciences, Guangzhou, 510080 China

**Keywords:** Urine proteome, Biomarkers, Behcet’s disease uveitis, Tandem mass tags, Data-independent acquisition

## Abstract

**Purpose:**

Behçet’s disease-associated uveitis (BDU) is a severe, recurrent inflammatory condition affecting the eye and is part of a systemic vasculitis with unknown etiology, making biomarker discovery essential for disease management. In this study, we intend to investigate potential urinary biomarkers to monitor the disease activity of BDU.

**Methods:**

Firstly, label-free data-dependent acquisition (DDA) and tandem mass tag (TMT)-labeled quantitative proteomics methods were used to profile the proteomes of urine from active and quiescent BDU patients, respectively. For further exploration, the remaining fifty urine samples were analyzed by a data-independent acquisition (DIA) quantitative proteomics method.

**Results:**

Twenty-nine and 21 differential proteins were identified in the same urine from BDU patients by label-free DDA and TMT-labeled analyses, respectively. Seventy-nine differentially expressed proteins (DEPs) were significantly changed in other active BDU urine samples compared to those in quiescent BDU urine samples by IDA analysis. Gene Ontology (GO) and protein-protein interaction (PPI) analyses revealed that the DEPs were associated with multiple functions, including the immune and neutrophil activation responses. Finally, seven proteins were identified as candidate biomarkers for BDU monitoring and recurrence prediction, namely, CD38, KCRB, DPP4, FUCA2, MTPN, S100A8 and S100A9.

**Conclusions:**

Our results showed that urine can be a good source of biomarkers for BDU. These dysregulated proteins provide potential urinary biomarkers for BDU activity monitoring and provide valuable clues for the analysis of the pathogenic mechanisms of BDU.

**Supplementary Information:**

The online version contains supplementary material available at 10.1186/s12886-024-03557-9.

## Introduction

Uveitis is a group of inflammatory eye diseases and a major cause of irreversible blindness among the working-age population [1]. It is mainly classified as infectious and noninfectious uveitis based on its etiology [2]. The majority (approximately 80%) of uveitis entities are noninfectious, resulting from autoimmune or autoinflammatory mechanisms [3]. Behcet’s disease (BD) is a chronic multisystemic autoimmune inflammatory disease involving the mucocutaneous, articular, ocular, digestive and central nervous systems. Ocular involvement, mainly presented as uveitis (Behcet’s disease-associated uveitis, BDU) [4], is the major cause of morbidity, with male being more commonly and severely involved [5]. Posterior segment is more commonly involved in BDU (50–93%) and is responsible for severe retinal damage and permanent vision loss, while the prevalence of anterior uveitis is only around 10% [6]. BDU is also one of the most common uveitis entities in the Chinese population, about 15.3% of all uveitis in the north China and 16.5% all over the country [7].

BDU is characterized by periods of relapse and remission. Recurrent episodes of uveitis attack may lead to irreversible vision loss due to sight-threatening complications, such as cystoid macular edema (CME), optic atrophy and retinal atrophy [8, 9]. The key points for the management of BDU are to control acute inflammation as soon as possible and to prevent recurrence [10]. The traditional treatment for BDU is glucocorticoid steroids (GCs) in combination with immunosuppressants (such as cyclosporine A and azathioprine); this treatment has a high acute remission rate but is less satisfactory for long-term control of remission. Biological agents, including interferon (IFN)-α, anti-tumor necrosis factor (TNF)-α agents (infliximab, adalimumab, etc.) are increasingly recognized as first-line treatments for BDU [11].

Because uveitis recurrence is seldom preceded by distinctive prodrome, it is urgent to identify biomarkers for disease monitoring, recurrence prediction and treatment evaluation for BDU. Indeed, a variety of potential blood-based biomarkers, including inflammatory and immunological [12–16], genetic [16–21], pathogenic, neutrophilic-associated, endothelial and coagulating factor-associated biomarkers, have been studied; the clinical application of these biomarkers requires further verification and validation [22]. Urine is an ultrafiltrate of the blood and a promising source for biomarkers [23]. Compared with other body fluids, urine can be obtained noninvasively, and its components are simpler and more stable [24, 25]. Recently, proteomic techniques have been successfully used to identify urinary biomarkers for chronic inflammatory diseases, including rheumatic arthritis (RA), psoriatic arthritis, osteoarthritis (OA) and inflammatory bowel disease (IBD) [26, 27]. Proteomics offers an expansive and dynamic view of the protein landscape, reflecting real-time changes in the disease state. The complexity and variability of BDU, characterized by episodic flares and remissions, make it a suitable candidate for proteomic studies as these can capture the transient biological changes associated with disease activity more effectively than static genetic markers.

The present study aimed to identify a panel of candidate protein biomarkers related to BDU in urine. In the discovery phase, label-free data-dependent acquisition (DDA) and tandem mass tags (TMT)-labeled quantitative proteomics techniques were used to profile the proteome of urine from active and quiescent BDU patients. In the validation phase, the data-independent acquisition (DIA) quantitative proteomics technique was used to analyze the remaining urine samples.

## Materials & methods

### Patients

Active or quiescent BDU patients treated at our center between January 2017 and July 2018 were included. The diagnosis of BD was based on the criteria of the International Study Group (ISG) or International Criteria for Behcet’s Disease (ICBD) [28]. The inclusion criteria were as follows: (1) 65 ≥ age ≥ 18 years old and (2) BDU presented as posterior or panuveitis. Criteria for active BDU include: (1) acute decrease in vision, pain or redness of eyes; and (2) fresh retinal bleeding, retinal arterial sheathing or retinal infiltrate; (3) increase in vitreous opacity by ≥ 1+ (Nussenblatt scale). Patients who fulfilled none of the inclusion criteria of active BDU for at least 3 months were defined as quiescence.

The exclusion criteria were as follows: (1) comorbidity with other systemic diseases, including cardiovascular diseases, diabetes, neurological disorders and other autoimmune disorders; (2) presence of local or systemic infections, such as acute conjunctivitis, keratitis, scleritis; (3) severe involvement of other vital systems/organs, including the central nervous system, cardiovascular system, and gastrointestinal tract; (4) presence of secondary glaucoma, severe cataracts or other complications; (5) extensive peripheral anterior synechia of the iris; (6) a past medical history of other severe ocular disorders, ocular trauma or surgeries; and (7) women in the menstrual period, medication, proteinuria.

The consent procedure and the study protocol were approved by the Institutional Review Board of the Peking Union Medical College Hospital, Chinese Academy of Medical Sciences (Project No. JS-1886). And all the methods were performed in accordance with the relevant guidelines and regulations of Institutional Review Board of the Peking Union Medical College Hospital, Chinese Academy of Medical Sciences. It was confirmed that verbal informed consent was acquired from every volunteer.

### Urine sample preparation

Every patient was asked to collect 30 ml of midstream of the second morning urine, and then stored at -80 °C. Urinary proteins were extracted from sixty individual urine samples (10 ml from each sample) by ethanol precipitation [29]. Ethanol was added, and precipitated at 4 °C for 12 h. After centrifugation, lysis buffer (8 mol/L urea, 2 mol/L thiourea, 50 mmol/L Tris, and 25 mmol/L DTT) was added to redissolve the precipitates. The proteins (100 µg) were then digested using trypsin (Promega, USA) following the standard FASP protocol [30].

### Peptide TMT labeling and offline HPLC separation

Ten peptide samples were individually labeled with Tandem Mass Tag Label Reagents according to the manufacturer’s protocol (Thermo Fisher Scientific, Germany, Lot number: RD231322). After the peptides were labeled with isobaric tags, they were mixed. High-pH reversed-phase fractionation chromatography was carried out using a Waters 2690 HPLC system. The details were described as previously [31]. The labeled peptide mixture was loaded onto XBridge C18 columns [32].

### Reversed-phase fractionation spin column separation

Pooled peptide samples were fractionated by a high-pH reversed-phase peptide fractionation kit (Thermo Pierce, USA) as previously described [33]. Briefly, pooled peptides were loaded onto the spin column. A step gradient of increasing acetonitrile concentrations (5, 7.5, 10, 12.5, 15, 17.5, 20 and 50% acetonitrile) was used to elute the bound peptides.

### LC-MS/MS setup for DDA and TMT analysis

The unlabeled peptide samples and TMT-labeled peptide fractions were analyzed by LC-MS/MS. Dissolved peptide sample was loaded onto a C18 trap column (75 μm × 2 cm, 3 μm, 100 Å), EASY-nLC 1200 HPLC system (Thermo Scientific, Germany). The 60 min 5–30% eluted gradient (flow rate 0.3 µl/min) was used for label-free and TMT-labeled analyses. The eluted peptides were analyzed by an Orbitrap Fusion Lumos Tribrid Mass Spectrometer (Thermo Scientific, Germany). The MS was setup as previously described [31].

### LC-MS/MS setup for DIA analysis

For both the DDA and DIA analyses, the same Orbitrap Fusion Lumos Tribrid Mass Spectrometer was employed. For the generation of the spectral library, ten fractions from the spin column were analyzed in DDA-MS mode. For the DIA-MS method, fifty individual samples were analyzed in DIA mode as previously described [34]. The parameters for the HPLC system were set as described in Sect. 2.6, and the eluted gradient was set to 90 min. For MS acquisition, 34 windows were developed.

### Label-free DDA and TMT-labeled quantitative analysis

The label-free MS data were analyzed by Mascot software (version 2.5.1, Matrix Science, UK) and Progenesis software (version 4.1, Nonlinear, Newcastle upon Tyne, UK), as previously described [33, 35]. False discovery rate (FDR) was set to 1%.

The TMT-labeled MS data were searched by Proteome Discoverer (version 2.3; Thermo Fisher Scientific, San Jose, CA, USA) with Sequest HT against the SwissProt_Homo sapiens database (released in May 2019, containing 20,358 sequences) as previously described [31]. False discovery rate (FDR) l was set to 1%.

### Label-free DIA quantitative analysis

The fractions’ raw data files acquired by the DDA mode were processed using Proteome Discoverer to generate the spectral library, for further DIA analysis. The search parameters were set as described before [36].

The MS files of DIA were imported to Spectronaut Pulsar with the default settings. In brief, For the extracted ion chromatogram (XIC) extraction window, a dynamic window and a nonlinear iRT calibration strategy were used. Cross-run normalization and a local normalization strategy was used [37]. Protein inference was performed on the principle of parsimony using the ID picker algorithm [38]. Q value cutoff was set as 0.01 (corresponding to an FDR of 1%). The peak areas of their respective fragment ions were calculated for peptide intensity.

### Bioinformatics and statistical analysis

Bioinformatics analysis was carried out to better study the biological function of the dysregulated proteins. GO analysis was performed on the 115 differential urinary proteins (http://www.geneontology.org/) [39, 40]. STRING database (http://www.string-db.org) were used to constructed the Protein-protein interaction networks as previously described [33, 34].

## Results & discussion

### BDU patients and urine samples

**Fig. 1:**
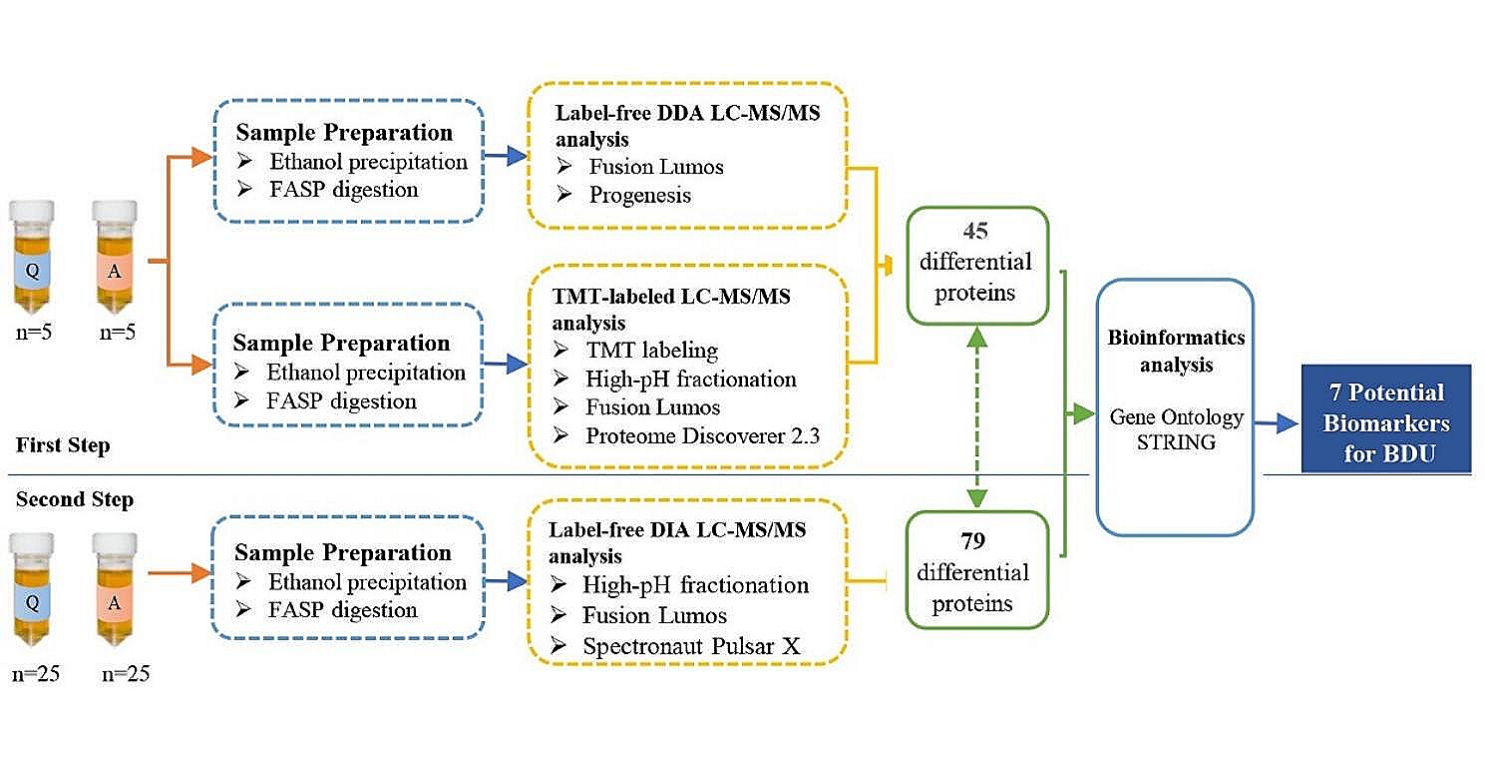
Workflow of urine proteomic study in BDU patients (Q: BDU patients in the quiescent phase; A: BDU patients in the active phase)

This is a retrospective research analysis. A summary of the overall experimental approach is presented in Fig. 1. In total, 62 BDU patients being treated at our center between January 2017 and July 2018 were included. Among these 62 urine samples, 2 were excluded due to protein degradation, and 60 were processed for LC-MS/MS analysis. Thirty patients were in the active stage, aged 29.4 ± 5.7 years, among which 21 were male; the other thirty patients were in the quiescent stage, aged 32.6 ± 8.9 years, among which 19 were male.

### Urine proteome differential proteins by label-free and TMT-labeled LC-MS/MS

In the label-free LC-MS/MS analysis, Mascot software was used for the database search, and then Progenesis software was used for quantification based on feature intensity. In total, 1 843 proteins with ≥ 1 unique peptides were identified with an FDR ≤ 1% at the protein level. All identification and quantification details of the 1 843 proteins are listed in supporting Table S1. Compared to the quiescent group, 29 urinary proteins in active samples were identified to have significantly differential abundance (1.5-fold change, *p* < 0.05) (Table 1).

**Table 1:** Details of the dysregulated proteins identified by label-free DDA analysis

UniProt ID	Protein name	FC	*P* value
Q9NQ79	Cartilage acidic protein 1	5.28	2.7E-03
P28907	ADP-ribosyl cyclase hydrolase 1	4.02	4.7E-02
P01742	Immunoglobulin heavy variable 1–69	3.88	3.5E-03
P02675	Fibrinogen beta chain	3.64	1.1E-02
P05165	Propionyl-CoA carboxylase alpha chain, mitochondrial	3.24	2.8E-02
P01880	Ig delta chain C region	3.08	2.3E-02
O15382	Branched-chain-amino-acid aminotransferase, mitochondrial	3.05	2.8E-02
P63000	Ras-related C3 botulinum toxin substrate 1	3.00	2.7E-03
P00915	Carbonic anhydrase 1	2.96	1.5E-02
P01024	Complement C3	2.62	1.8E-02
P02792	Ferritin light chain	2.67	3.7E-02
Q13621	Solute carrier family 12 member 1	2.58	2.5E-03
P19801	Amiloride-sensitive amine oxidase [copper-containing]	2.46	5.1E-05
P02748	Complement component C9	2.31	2.6E-03
P54108	Cysteine-rich secretory protein 3	2.26	2.4E-02
P58546	Myotrophin	2.21	2.8E-02
P01860	Ig gamma-3 chain C region	2.20	3.2E-02
P00450	Ceruloplasmin	2.15	3.3E-02
P12277	Creatine kinase B-type	2.11	1.3E-03
P00568	Adenylate kinase isoenzyme 1	2.10	9.4E-03
Q9BTY2	Plasma alpha-L-fucosidase	1.53	3.0E-02
P27487	Dipeptidyl peptidase 4	1.50	1.9E-02
Q5TFQ8	Signal-regulatory protein beta-1 isoform 3	-2.02	1.4E-02
Q5JXA9	Signal-regulatory protein beta-2	-2.07	6.6E-03
Q8N386	Leucine-rich repeat-containing protein 25	-2.22	1.3E-02
P35052	Glypican-1	-2.49	4.3E-02
Q6XQN6	Nicotinate phosphoribosyltransferase	-2.71	7.0E-03
Q99795	Cell surface A33 antigen	-3.09	1.0E-02
Q03167	Transforming growth factor beta receptor type 3	-3.36	3.2E-02

In the TMT-labeled LC-MS/MS analysis, the quantification was based on the reporter using Proteome Discoverer. In total, 2 466 proteins with ≥ 1 unique peptides were identified with FDR ≤ 1% at the protein level. All identification and quantification details of the 2 466 proteins are listed in supporting Table S2. Compared to the quiescent group, 21 urinary proteins in active samples were identified to have significantly differential abundance (1.5-fold change, *p* < 0.05) (Table 2).

**Table 2:** Details of the dysregulated proteins identified by TMT-labeled analysis

UniProt ID	Protein name	FC	*P* value
P23284	Peptidyl-prolyl cis-trans isomerase B	2.00	4.1E-02
P30479	HLA class I histocompatibility antigen, B alpha chain	1.94	2.7E-02
Q9NQ79	Cartilage acidic protein 1	1.79	1.7E-03
P01036	Cystatin-S	1.68	4.1E-02
P02792	Ferritin light chain	1.63	5.0E-02
O75947	ATP synthase subunit d, mitochondrial	1.53	8.3E-03
P01780	Immunoglobulin heavy variable 3–7	1.52	7.1E-03
P05997	Collagen alpha-2	1.51	4.9E-02
P63000	Ras-related C3 botulinum toxin substrate 1	1.89	1.3E-02
P01860	Immunoglobulin heavy constant gamma 3	1.56	6.9E-03
Q03167	Transforming growth factor beta receptor type 3	-1.50	4.7E-02
P12081	Histidine–tRNA ligase, cytoplasmic	-1.51	4.7E-02
P37837	Transaldolase	-1.57	1.6E-02
P26447	Protein S100-A4	-1.58	1.2E-02
Q15847	Adipogenesis regulatory factor	-1.58	3.8E-02
O75487	Glypican-4	-1.59	1.0E-03
Q13838	Spliceosome RNA helicase DDX39B	-1.65	9.1E-03
P06702	Protein S100-A9	-1.72	4.5E-02
P01225	Follitropin subunit beta	-1.78	3.2E-02
P25815	Protein S100-P	-1.88	1.2E-02
P05109	Protein S100-A8	-1.90	4.0E-02

- Means decreasing trend

### Urine proteome differential proteins by label-free DIA LC-MS/MS

Fifty urine samples were analyzed by the LC-MS/MS workflow. A total of 1 676 proteins with at least one unique peptide with FDR < 1% at protein level was identified, and all identification and quantification details are listed in supporting Table S3. Seventy-nine proteins were significantly changed in active BDU urine samples compared to the proteins in quiescent BDU urine samples (Table S4).

**Fig. 2:**
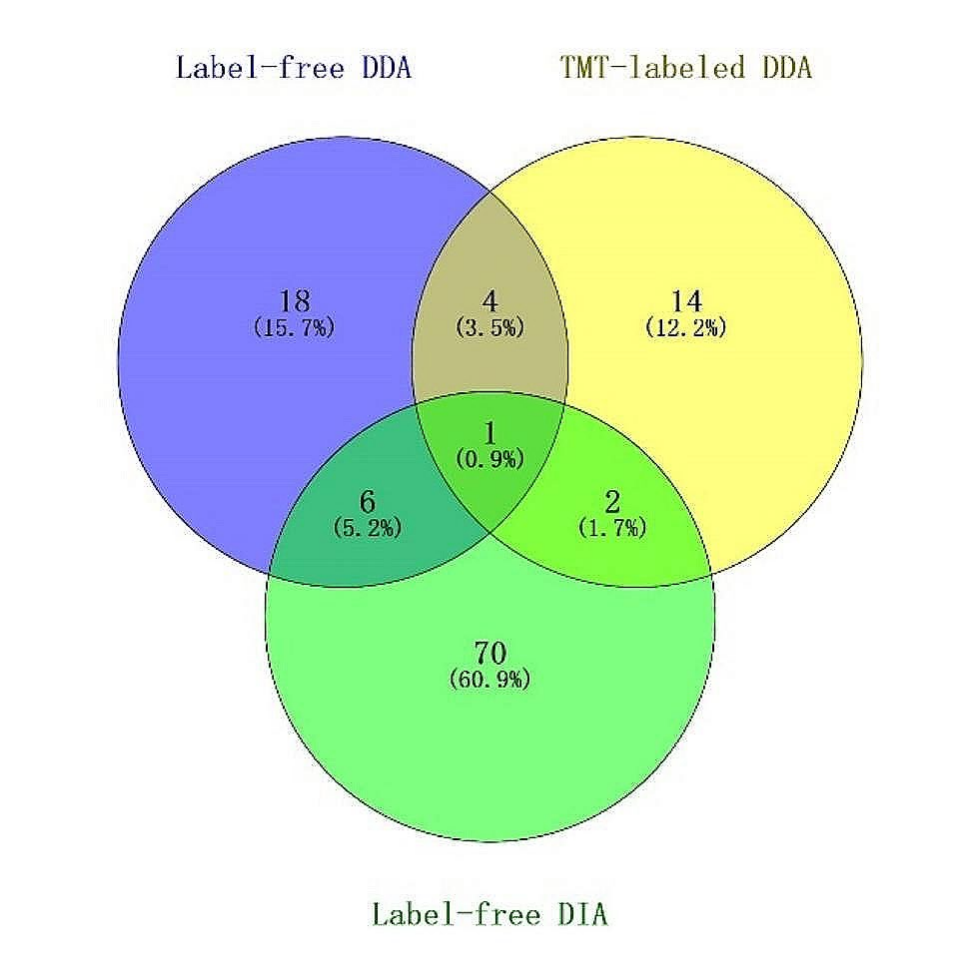
Vein diagram of the differential urinary proteins in BDU patients identified by label-free DDA, TMT-labeled DDA, and label-free DIA LC-MS/MS methods

Combined the results from these three proteomic methods, a total of 115 urinary proteins changed significantly (Fig. 2). Five differential proteins were both identified by label-free and TMT-labeled DDA methods. Nine differential proteins in the first 10 subjects identified by label-free and TMT-labeled DDA methods, were also candidates in the label-free DIA methods. Among, 7 DEPs had consistent expression trend, including CD38, KCRB, DPP4, FUCA2, MTPN, S100A8 and S100A9.

### Gene ontology analysis

**Fig. 3:**
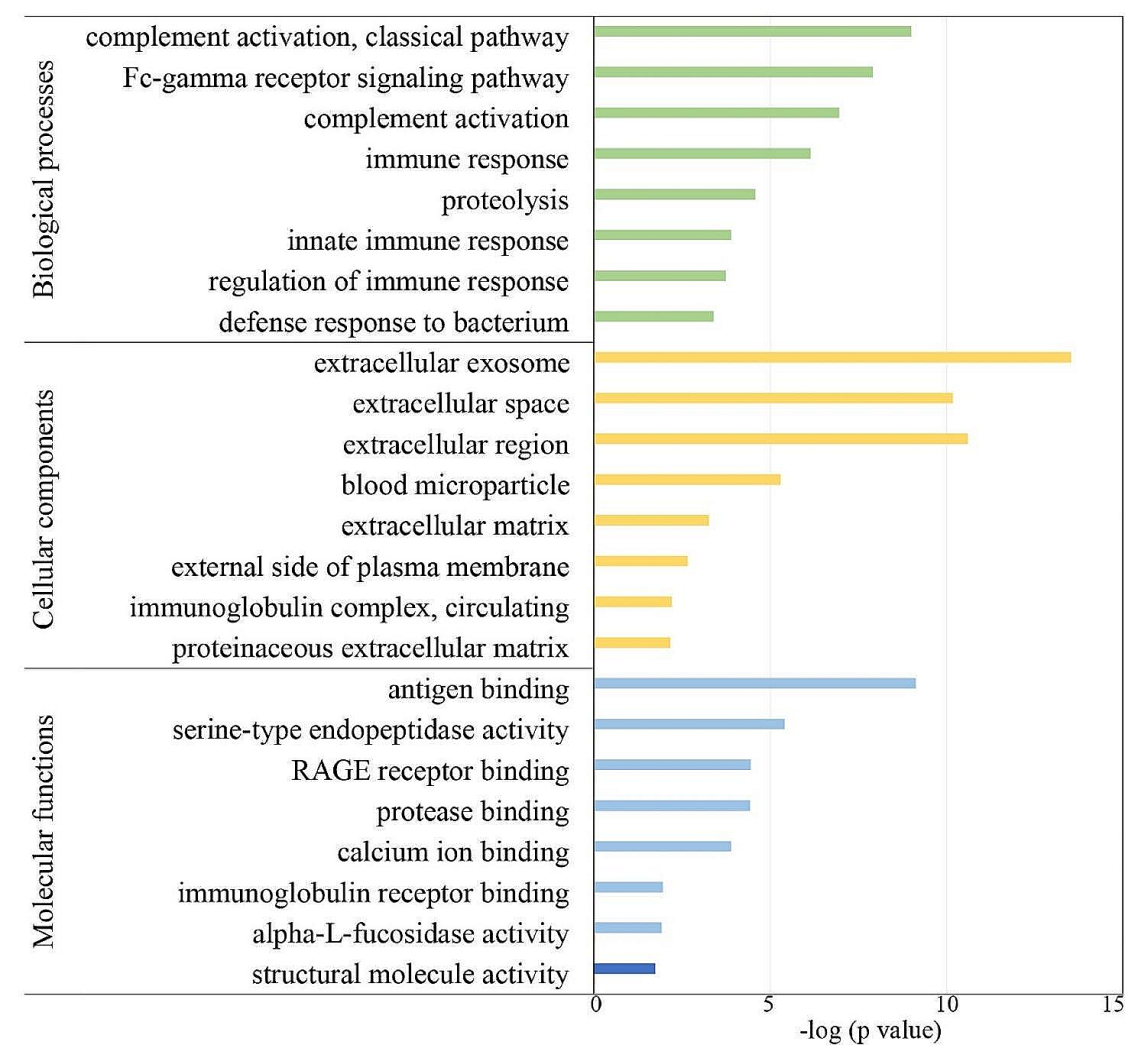
GO enrichment analysis of the differential proteins in BDU patients

The GO functional annotation was performed on the 115 differentially expressed proteins. All differential proteins were annotated and classified to be involved with certain biological processes (Fig. 3).

GO enrichment analysis showed that the immune response, complement activation, Fc-gamma receptor signaling pathway and proteolysis were the main biological processes involved. Differential proteins in these GO terms include FUCA2, CD38, DPP4. Previously results indicate that activated innate immunity plays an important role in the pathogenesis of BD [41, 42]. In the cellular component category, most of these dysregulated proteins were extracellular exosomes, extracellular space and extracellular region proteins. In the molecular function category, antigen binding, serine-type endopeptidase activity, calcium ion binding, RAGE receptor binding, and protease binding were overrepresented.

### Protein-protein interaction network

**Fig. 4:**
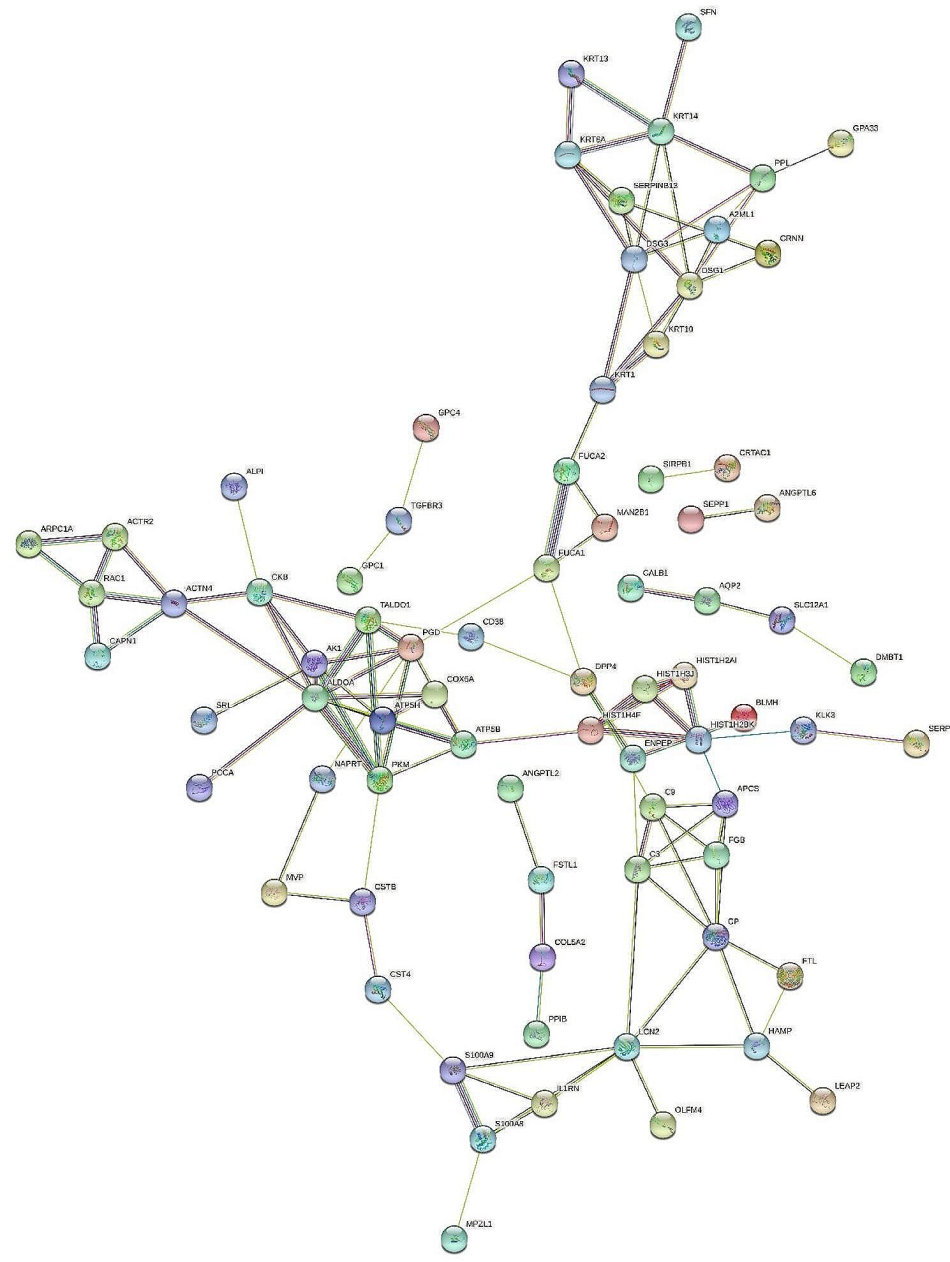
STRING PPI network analysis of the differential proteins in BDU patients. The average local clustering coefficient is 0.441, average node degree is 2.29, and *p*-value < 1.0e-16

To further discover the pathogenic mechanisms in BDU, the protein-protein interaction (PPI) network for the 115 differential proteins was constructed by STRING (Fig. 4). The STRING PPI network analysis indicated that the average local clustering coefficient is 0.441, average node degree is 2.29, and *p*-value is less than 1.0e-16. This reveals that these differential proteins were closely biologically connected. As shown in Fig. 4, several key proteins, including FUCA2, PGD, C3, DPP4, and S100A9, are centrally located within the protein-protein interaction network. This central placement suggests that these proteins could play significant roles in regulating molecular pathways involved in the pathogenesis of BDU.

## Discussion

In this preliminary study, a total of 115 differential urinary proteins (DEPs) were identified by three proteomic methods. Nine differential proteins in the first 10 subjects identified by label-free and TMT-labeled DDA methods, were also candidates in the label-free DIA methods. Among, 7 DEPs had consistent expression trend, including CD38, KCRB, DPP4, FUCA2, MTPN, S100A8 and S100A9 (Table 3), which hold the potentials for BDU monitoring and recurrence prediction. Of these, several proteins have been used as disease markers. Further study on these differential proteins is expected to deepen the role of these proteins in the pathogenesis of BDU.

**Table 3:** The potential urinary proteins for monitoring recurrence of BDU

UniProt Ids	Protein names	DIA		DDA and TMT	
		FC	*p* value	FC	*p* value
P28907	ADP-ribosyl cyclase 1	1.64	3.0E-02	4.02	4.7E-02
P58546	Myotrophin	1.73	5.1E-04	2.21	2.8E-02
P12277	Creatine kinase B-type	1.73	3.5E-01	2.11	1.3E-03
Q9BTY2	Plasma alpha-L-fucosidase	1.68	1.3E-02	1.53	3.0E-02
P27487	Dipeptidyl peptidase 4	1.98	1.2E-02	1.50	1.9E-02
P05109	Protein S100-A8	-5.25	4.6E-02	-1.90	4.0E-02
P06702	Protein S100-A9	-5.98	3.0E-02	-1.72	4.5E-02

ADP-ribosyl cyclase 1 (CD38) was initially found on thymocytes and T lymphocytes and was distributed throughout the immune system. CD38 is a multifunctional molecule involved in health and diseases, such as chronic lymphocytic leukemia, myeloma and ovarian carcinoma [43]. The expression of CD38 was higher in colon specimens from patients with Crohn’s disease and ulcerative colitis than that from normal controls [44]. In several mouse models for autoimmunity and immunopathology, CD38-/- mice demonstrate an ameliorated course in several diseases, such as collagen-induced arthritis model, allergen-induced airway hyperresponsiveness model, and DSS-induced colitis model [45–47].

Dipeptidyl peptidase 4 (DPP4) is a cell surface glycoprotein receptor involved in the costimulatory signal essential for T-cell receptor-mediated T-cell activation. Previous studies have shown that compared with normal controls, patients with RA, systemic lupus erythematosus, systemic sclerosis and IBD have decreased levels of serum DPP4 [48–50]. Conversely, T cell surface expression of DPP4 is higher in RA patients than that in normal controls.

Plasma alpha-L-fucosidase (FUCA2) is a hydrolase that plays a key role in the pathogenesis of glycoprotein lysosomal storage disorders [51]. The role of FUCA2 in inflammatory processes and autoimmune pathologies is well documented [52]. High urinary FUCA2 levels were observed in pediatric patients with type 1 diabetes [53]. Low plasma FUCA2 levels were observed in patients with chronic autoimmune disorders, such as Sjögren syndrome. In addition, it also gained importance as potential serological markers in some forms of cancer, such as hepatocellular carcinoma [54].

S100A8 and S100A9 have been classified as EF hand calcium-binding proteins belonging to the S100 protein family. S100A8/A9 are granulocyte and monocyte specific and play a prominent role in a variety of pathological processes, such as inflammation, infection, and autoimmune diseases [55, 56]. S100A8/A9 plasma levels were significantly elevated in uveitis patients compared to non-uveitic controls [56]. Higher expression of S100A8, but lower expression of S100A9 were found in tears from children with juvenile idiopathic arthritis associated uveitis (JIA-U) compared to those from idiopathic chronic anterior uveitis (I-CAU) [57]. There is research showing that there is no significant difference in the expression level of S100A8 between the active and quiescent phases of BDU. Our study is distinct in that it analyzes urinary proteins and healthy subjects were not included. It is conceivable that the urinary excretion patterns of these proteins do not directly mirror serum levels or tissue expression, possibly due to renal processing or the specific dynamics of protein shedding into urine.

In our study, we focused on the identification of urinary biomarkers to monitor the activity of BDU. Given the systemic nature of Behçet’s disease, which can affect multiple organs and systems beyond the eyes, the specificity of urinary proteins as biomarkers for ocular activity poses a significant challenge. In the selection of our patient cohort, we applied rigorous criteria to focus primarily on individuals exhibiting primarily ocular manifestations. Unfortunately, detailed clinical data regarding the activity of Behçet’s disease in other systems (e.g., vascular, gastrointestinal, neurological) were not comprehensively available for all participants, which restricts our ability to differentiate the source of protein alterations observed in the urine. As such, this limitation should be considered when interpreting the results of our study. We recommend that future studies include detailed systemic evaluations and consider using organ-specific biomarkers in conjunction with urinary.

Our results showed that urine can be a good source of biomarkers for BDU. These dysregulated proteins provide potential urinary biomarkers for BDU activity monitoring and provide valuable clues for the analysis of the pathogenic mechanisms of BDU.

### Electronic supplementary material

Below is the link to the electronic supplementary material.

Supplementary Material 1

## Data Availability

All data generated or analyzed during this study are included in this published article and the Supplementary information files.
